# Decreased muscle‐derived musclin by chronic resistance exercise is associated with improved insulin resistance in rats with type 2 diabetes

**DOI:** 10.14814/phy2.14823

**Published:** 2021-05-05

**Authors:** Mio Shimomura, Naoki Horii, Shumpei Fujie, Kenichiro Inoue, Natsuki Hasegawa, Keiko Iemitsu, Masataka Uchida, Motoyuki Iemitsu

**Affiliations:** ^1^ Faculty of Sport and Health Science Ritsumeikan University Kusatsu Shiga Japan; ^2^ Research Fellow of Japan Society for the Promotion of Science Chiyoda‐ku Tokyo Japan; ^3^ Research Organization of Science and Technology Ritsumeikan University Kusatsu Shiga Japan

**Keywords:** insulin resistance, musclin, resistance exercise, type 2 diabetes

## Abstract

Chronic resistance exercise induces improved hyperglycemia in patients with type 2 diabetes mellitus. Musclin, a muscle‐derived secretory factor, is involved in the induction of insulin resistance via the downregulation of the glucose transporter‐4 (GLUT‐4) signaling pathway in skeletal muscles. However, whether musclin affects the mechanism of resistance exercise remains unclear. This study aimed to clarify whether decreased muscle‐derived musclin secretion in chronic resistance exercise is involved in the improvement of insulin resistance via the GLUT‐4 signaling pathway in rats with type 2 diabetes. Male, 20‐week‐old, Otsuka Long‐Evans Tokushima Fatty (OLETF) rats, a type 2 diabetes model, were randomly divided into two groups: sedentary control (OLETF‐Con) and chronic resistance exercise (OLETF‐RT; climbing a ladder three times a week on alternate days for 8 weeks), whereas Long‐Evans Tokushima Otsuka rats were used as the nondiabetic sedentary control group. OLETF‐Con rats showed increased fasting glucose levels, decreased insulin sensitivity index (QUICKI), muscle GLUT‐4 translocation, and protein kinase B (Akt) phosphorylation, and concomitantly increased muscle musclin expression. In contrast, OLETF‐RT rats significantly reduced muscle musclin expression, improved hyperglycemia, and QUICKI through an accelerated muscle GLUT‐4/Akt signaling pathway. Moreover, chronic resistance exercise‐induced reduction of muscle musclin was correlated with changes in fasting glucose, QUICKI, GLUT‐4 translocation, and Akt phosphorylation. These findings suggest that the reduction in muscle‐derived musclin production by chronic resistance exercise may be involved in improved insulin resistance in rats with type 2 diabetes.

## Graphical abstract

8‐week resistance exercise significantly reduced musclin protein levels in the skeletal muscle of rats with type 2 diabetes. Importantly, chronic resistance exercise‐induced reduction of musclin protein levels in the skeletal muscle were significantly associated with activation of muscle Akt phosphorylation and GLUT4 translocation. These results suggest that the chronic resistance exercise‐induced reduction of musclin is involved in the improvement of hyperglycemia and insulin resistance via the activation of the muscle Akt/GLUT4 signaling pathway in rats with type 2 diabetes.

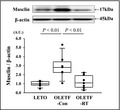

## 1 INTRODUCTION

Type 2 diabetes is increasingly prevalent worldwide, affecting 1 in 11 adults (International Diabetes Federation, 2019). The beneficial effects of regular aerobic and resistance exercises for patients with type 2 diabetes, which include improved glycemic control and reduced insulin resistance, have been well established by many epidemiological studies and meta‐analyses (Snowling & Hopkins, 2006; Umpierre et al., 2011; Yang et al., 2014). Chronic resistance exercise induced muscle hypertrophy and reduced fasting glucose, glycosylated hemoglobin, and the insulin resistance index in type 2 diabetes patients in randomized controlled studies (Arora et al., 2009; Bacchi et al., 2012; Dunstan et al., 2002; Kadoglou et al., 2013; Sigal et al., 2007). As part of the molecular mechanism underlying the effects of chronic resistance exercise, an increase in the uptake and utilization of glucose, via increases in the transcription and translocation of glucose transporter 4 (GLUT‐4) in the skeletal muscle, contributes to the improvement of glycemic control (Horii et al., 2016; Kim et al., 2015).

It is well known that the muscle‐derived secretory factor “myokine” regulates energy metabolism homeostasis by releasing a variety of bioactive factors (Severinsen & Pedersen, 2020). Musclin, a myokine, is a 130‐amino acid peptide that is mainly expressed in the skeletal muscles of rodents (Nishizawa et al., 2004). Nishizawa et al. (2004) demonstrated that musclin mRNA expression was augmented in the gastrocnemius muscles in mice with type 2 diabetes. In addition, musclin treatment suppresses insulin‐stimulated 2‐deoxy‐D‐[1‐^3^H] glucose uptake in C2C12 cells in vitro (Nishizawa et al., 2004). Furthermore, musclin inhibits insulin‐stimulated protein kinase B (Akt) mRNA expression and Akt phosphorylation in ex vivo cultures of rat muscle tissues (Liu et al., 2008). Muscle GLUT‐4 translocation is accelerated via the activation of insulin‐stimulated phosphatidylinositol 3‐kinase and Akt phosphorylation (Krook et al., 2004; Ryder et al., 2001). Therefore, muscle‐derived musclin is involved in insulin resistance in type 2 diabetes. In fact, in human and animal studies, the blood and muscle tissue levels of musclin are positively associated with fasting blood glucose levels and the insulin resistance index in patients with obesity and type 2 diabetes (Chen, Liu, Sui, Yang, et al., 2017; Chen, Liu, Sui, Zhang, et al., 2017). Recent studies have shown that chronic aerobic exercise reduces the basal mRNA and protein expression levels of musclin in the skeletal muscle of obese rats fed a high‐fat diet (Yu et al., 2016). Accordingly, a reduction in muscle‐derived musclin secretion may be involved in the mechanism through which resistance exercise leads to better glycemic control in patients with type 2 diabetes. However, it remains unclear whether the chronic resistance exercise‐induced changes in musclin secretion contribute to the improvement of glycemic control in type 2 diabetes.

In this study, we aimed to investigate whether a chronic resistance exercise‐induced decrease in musclin secretion in muscle is associated with an improvement in insulin resistance via the activation of the muscle GLUT‐4 signaling pathway in type 2 diabetes model rats. Therefore, to test our hypothesis, we subjected Otsuka Long‐Evans Tokushima Fatty (OLETF) rats with type 2 diabetes to resistance exercises for 8 weeks and evaluated the effects of resistance exercise‐induced changes in musclin on the insulin resistance index via the muscle Akt/GLUT‐4 regulated signaling pathway.

## 2 METHODS

### 2.1 Animals and protocol

The study design and all experimental procedures were approved by the Committee on Animal Care at Ritsumeikan University in Japan. Male OLETF rats (6‐week‐old) were obtained from Japan SLC (Shizuoka, Japan). All rats were maintained individually in an animal facility under a 12‐h light‐dark cycle; they were fed a standard diet (CE‐2: CLEA Japan, Kyoto, Japan) and provided access to water ad libitum. After 14 weeks, the 20‐week‐old OLETF rats were randomly assigned to sedentary control (OLETF‐Con) or resistance training (OLETF‐RT) groups (*n* = 7 in each group). Additionally, nondiabetic and aged‐matched Long‐Evans Tokushima Otsuka (LETO) rats were included in a healthy sedentary control group (*n* = 7). Post‐treatment experiments in the OLETF‐RT group were performed more than 48 h after the end of the last resistance exercise session to avoid the acute effect of exercise. Following an overnight fast for 12 h, and after measuring the body weight, blood samples were obtained from the abdominal aorta under general anesthesia. After sacrifice, the epididymal fat, heart, and gastrocnemius, soleus, and plantaris muscles were quickly removed, weighed, rinsed in ice‐cold saline, frozen in liquid nitrogen, and stored at −80°C until use.

### 2.2 Resistance‐training protocol

Resistance training was performed 3 days a week for 8 weeks using a climbing ladder (length: 1.1 m, grid step: 2.0 cm, and inclination: 80°) as described in a previous study (Horii et al., 2016; Hornberger & Farrar, 2004). The OLETF‐RT group climbed the ladder for three sets of four repetitions each, and the rats were allowed to rest for 1 min between each set.

### 2.3 Immunoblot analysis

Western blotting analysis was performed as reported previously (Horii et al., 2016, 2020; Sato et al., 2017). Briefly, the gastrocnemius muscle samples (40 µg of total protein) of containing mixed fast and slow fiber types were separated via 10% SDS‐PAGE and transferred to PVDF membranes (Merck Millipore). The membranes were blocked for 1 h with blocking buffer (5% skim milk in PBS with 0.1% Tween 20 [PBS‐T]) and incubated for more than 12 h in blocking buffer at 4°C with antibodies (diluted 1:1000 in blocking buffer) against musclin (sc‐99096; Santa Cruz Biotechnology), Ser473 phosphorylated Akt (#9271; Cell Signaling Technology), or total Akt (#9272; Cell Signaling Technology). β‐actin protein (#4967; Cell Signaling Technology) was used as an internal control. The membranes were washed three times with PBS‐T and incubated for 1 h at 22–24°C with horseradish peroxidase‐conjugated secondary antibody and anti‐rabbit immunoglobulins (GE Healthcare Biosciences, and Cell Signaling Technology) diluted 1:3000 in blocking buffer. The membranes were washed three times with PBS‐T. Finally, the levels of these protein were detected using the Enhanced Chemiluminescence Plus system (GE Healthcare Biosciences), visualized on an ImageQuant LAS 4000 imager (GE Healthcare Biosciences). Densitometry was performed using ImageJ 1.48 software (National Institutes of Health, Bethesda, MD). The musclin protein expression levels were normalized to β‐actin protein levels, and Akt phosphorylation expression levels were normalized to total Akt protein levels.

### 2.4 Preparation of cytosolic and plasma membrane protein fractions

To assess GLUT‐4 translocation, two different cellular fractions were used as in previous studies (Benomar et al., 2006; Horii et al., 2016; Sato et al., 2011, 2017). In brief, the gastrocnemius muscles were homogenized with buffer A containing 20 mM Tris (pH 7.4), 1 mM EDTA, 0.25 mM EGTA, 250 mM sucrose, 1 mM DTT, 50 mM NaF, 25 mM sodium pyrophosphate, and 40 mM β‐glycerophosphate. The resulting homogenates were centrifuged at 400 *g* for 15 min to remove debris. The supernatant was collected and centrifuged at 215,000 *g* for 1 h. The resulting supernatant was used as the cytosolic fraction. Pellets from different fractions were solubilized for 1 h in buffer B containing 20 mM Tris (pH 7.4), 1 mM EDTA, 0.25 mM EGTA, 2% Triton X‐100, 50 mM NaF, 25 µM sodium pyrophosphate, and 40 mM β‐glycerophosphate. Before being used as the plasma membrane fraction, the homogenate was centrifuged briefly, and the supernatant was centrifuged for 1 h at 215,000 *g*. The levels of GLUT‐4 protein (# 07–1404; Merck Millipore) were measured in both the cytosol and membrane proteins via western blotting analysis. Furthermore, the protein expression of sodium‐potassium ATPase (Na^+^/K^+^ ATPase: ab7671; Abcam) as a marker of the plasma membrane fractions in the membrane fraction and β‐actin as a cytosolic fraction marker were examined. The translocation of GLUT‐4 was evaluated based on the difference in protein levels in these fractions (Horii et al., 2016; Sato et al., 2011, 2017).

### 2.5 Fasting blood glucose and insulin levels

Fasting blood glucose levels were assessed using a blood glucose meter (Terumo Corporation) in blood obtained from the tail vein after the intervention period under the 12 h overnight fasting conditions. Serum insulin levels were measured using an enzyme‐linked immunosorbent assay kit (Shibayagi), and optical density at 450 nm was measured using a xMark microplate spectrophotometer (Bio‐Rad Laboratories).

### 2.6 Insulin sensitivity index

The quantitative insulin sensitivity check index (QUICKI), as an index of insulin sensitivity, was calculated according to a previous study on fasting blood glucose and insulin levels (Horii et al., 2016, 2019; Sato et al., 2012). QUICKI = 1/[log(I0) + log(G0)], where I0 is the fasting insulin (µU/ml), and G0 is the fasting glucose (mg/dl).

### 2.7 Histochemical analysis

The gastrocnemius muscle samples frozen for histochemical analysis were sliced into 10 µm‐thick sections in a cryostat at −20°C. The sections were subjected to hematoxylin and eosin staining and measurement of a cross‐sectional area (CSA) of the muscle fibers using ImageJ 1.48 software (National Institutes of Health) (Horii et al., 2016; Liu et al., 2014). At least five sections were taken from each sample, and 10 microscopic fields were examined at ×400 magnification using a Leica DFC425 microscope (Leica Microsystems).

### 2.8 Statistical analysis

All values are expressed as the mean ± SE. Statistical evaluations were analyzed using one‐way ANOVA. A post hoc comparison test was used to correct for multiple comparisons (Fisher's test) when the analyses showed significant differences. For ANOVA, *p* < 0.05 was considered significant. Relationships between musclin protein levels in skeletal muscle and fasting blood glucose levels, insulin levels, QUICKI, Akt phosphorylation, and GLUT‐4 translocation levels in skeletal muscle were determined using Pearson correlation coefficients. All analyses were performed using Stat View 5.0 (SAS Institute).

## 3 RESULTS

### 3.1 Animal characteristics

In the OLETF‐Con group, body weight and epididymal fat mass were significantly greater than those in the LETO group (*p* < 0.01, Table 1), whereas those in the OLETF‐RT group were significantly lower than those in the OLETF‐Con group (*p* < 0.05, Table 1). No significant difference in left ventricle mass was observed between the LETO and OLETF‐Con groups (Table 1). Left ventricle mass was significantly greater in the OLETF‐RT group than in the OLETF‐Con group (*p* < 0.01, Table 1). Additionally, the CSA of the gastrocnemius muscle, gastrocnemius muscle mass, soleus muscle mass, and plantaris muscle mass in the OLETF‐Con group were significantly lower than those in the LETO group (*p* < 0.01, Table 1), whereas the CSA of gastrocnemius muscle, gastrocnemius muscle mass, and soleus muscle mass were significantly greater in the OLETF‐RT group than in the OLETF‐Con group (*p* < 0.01, Table 1). In the OLETF‐RT group, the plantaris muscle mass tended to be greater than that in the OLETF‐Con group (*p* = 0.082, Table 1).

**TABLE 1:** Animal characteristics

	LETO	OLETF‐Con	OLETF‐RT
BW (g)	483.1 ± 11.7	627.3 ± 6.4**	587.9 ± 12.2
Epididymal fat mass (mg/g BW)	18.0 ± 0.5	25.0 ± 1.7**	21.1 ± 1.0^†^
Left ventricle mass (mg/g BW)	1.89 ± 0.08	1.86 ± 0.10	2.79 ± 0.13
Gastrocnemius muscle mass (mg/g BW)	4.90 ± 0.17	3.34 ± 0.09**	4.22 ± 0.08
Soleus muscle mass (mg/g BW)	0.39 ± 0.01	0.32 ± 0.01**	0.38 ± 0.01
Plantaris muscle mass (mg/g BW)	0.85 ± 0.02	0.70 ± 0.03*	0.77 ± 0.02
CSA of gastrocnemius muscle (cm^2^)	2050.0 ± 72.9	1385.7 ± 60.7**	1800.8 ± 34.3

Values are mean ± SE.

Abbreviations: BW, body weight; CSA, cross sectional area; LETO, healthy‐sedentary control group; OLETF‐Con, OLETF‐sedentary control group; OLETF‐RT, OLETF‐resistance training group.

* *p* < 0.05 vs. LETO,

** *p* < 0.01 vs. LETO,

† *p* < 0.05 vs. OLETF‐Con,

†† *p* < 0.01 vs. OLETF‐Con.

### 3.2 Fasting blood glucose, insulin concentrations, and insulinEffects of 8weeks of resistance training on fasting blood glucose sensitivity index

**FIGURE 1:**
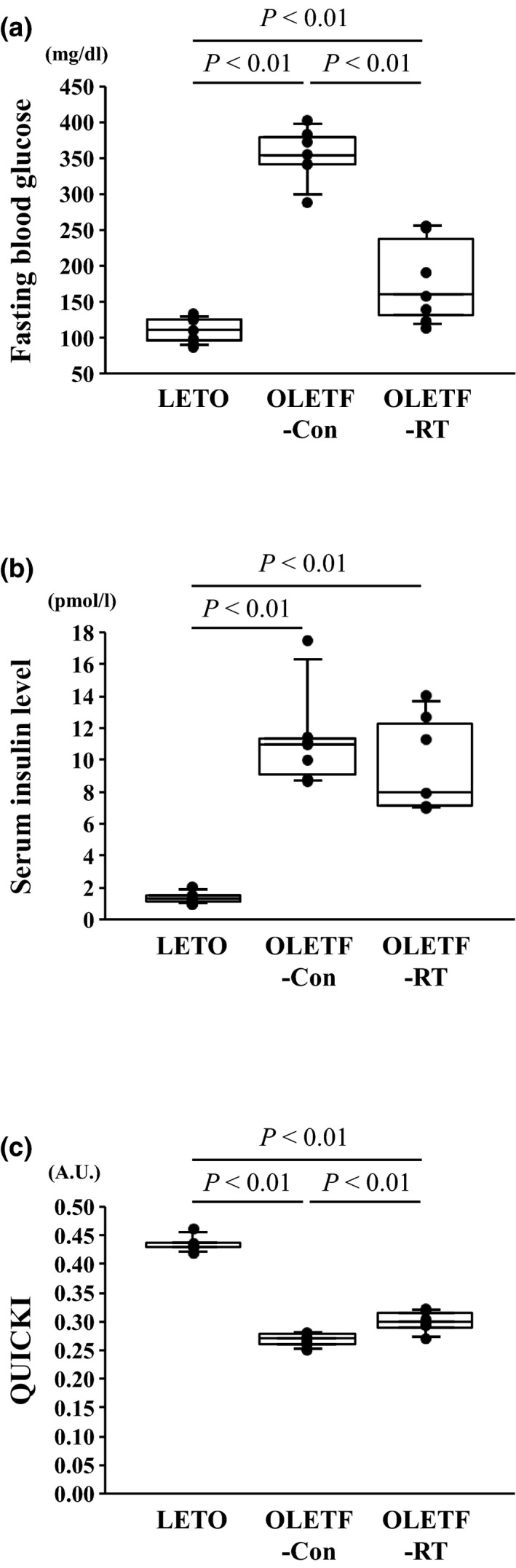
Effects of 8 weeks of resistance training on fasting blood glucose (a), insulin levels (b), and QUICKI (c). A.U., arbitrary units. Values are the mean ± SE. ●, individual values

In the OLETF‐Con group, fasting blood glucose and serum insulin levels were significantly higher than those in the LETO group (*p* < 0.01, Figure 1a,b), whereas fasting blood glucose levels were significantly lower in the OLETF‐RT group than in the OLETF‐Con group (*p* < 0.01, Figure 1a). However, there was no significant difference in insulin levels between the OLETF‐Con and OLETF‐RT groups (Figure 1b). In addition, QUICKI in the OLETF‐Con group was significantly lower than that in the LETO group (*p* < 0.01, Figure 1c), whereas QUICKI in the OLETF‐RT group was significantly greater than that in the OLETF‐Con group (*p* < 0.01, Figure 1c).

### 3.3 Musclin protein, Akt phosphorylation, and GLUT‐4 translocation in skeletal muscle

**FIGURE 2:**
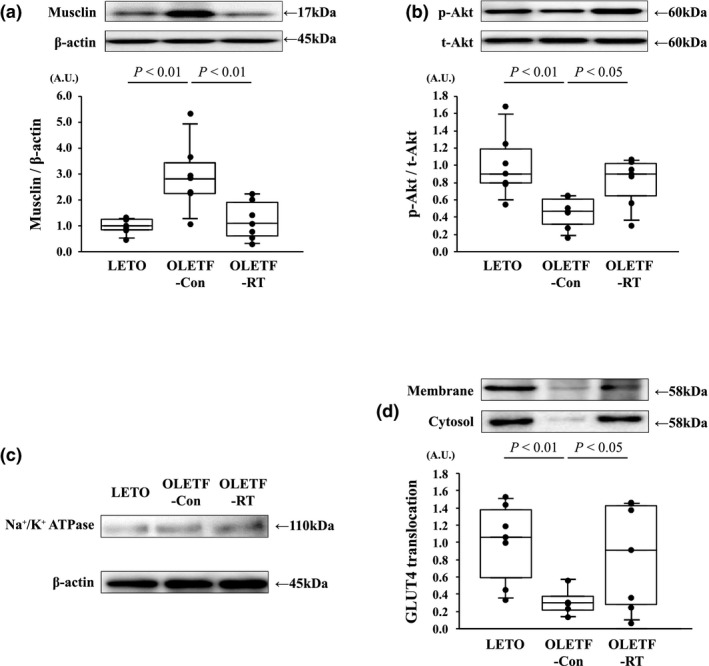
Effects of 8 weeks of resistance training on musclin protein (a), Akt phosphorylation at Ser473 (b), and GLUT‐4 translocation (d) levels in the gastrocnemius muscle. Representative immunoblotting image and histograms of the levels of musclin protein, Akt phosphorylation in GLUT‐4 in the cytosolic and membrane fractions are shown. Na^+^/K^+^ ATPase protein in the plasma membrane fractions and β‐actin protein in cytosolic fraction are shown (c). Musclin protein, Akt phosphorylation, and GLUT‐4 translocation levels are represented relative to each expression as fold‐changes from the gastrocnemius muscle in the LETO group. A.U., arbitrary units. Values are the mean ± SE. ●, individual values

In the OLETF‐Con group, muscle musclin protein levels were significantly greater than that in the LETO group (*p* < 0.01, Figure 2a), whereas muscle musclin protein levels in the OLETF‐RT group were significantly lower than those in the OLETF‐Con group (*p* < 0.01, Figure 2a). Additionally, muscle Akt phosphorylation levels in the OLETF‐Con group were significantly lower than those in the LETO group (*p* < 0.01, Figure 2b), whereas muscle Akt phosphorylation levels in the OLETF‐RT group were significantly greater than those in the OLETF‐Con group (*p* < 0.05, Figure 2b). To assess GLUT4 translocation, we confirmed the Na^+^/K^+^ ATPase protein in plasma membrane fractions and β‐actin protein in the cytosolic fractions (Figure 2c). Additionally, muscle GLUT‐4 translocation levels in the OLETF‐Con group were significantly lower than those in the LETO group (*p* < 0.01, Figure 2d), whereas muscle GLUT‐4 translocation levels in the OLETF‐RT group were significantly greater than those in the OLETF‐Con group (*p* < 0.05, Figure 2d).

### 3.4 Relationships between musclin protein level in skeletal muscle and glucose metabolism

**FIGURE 3:**
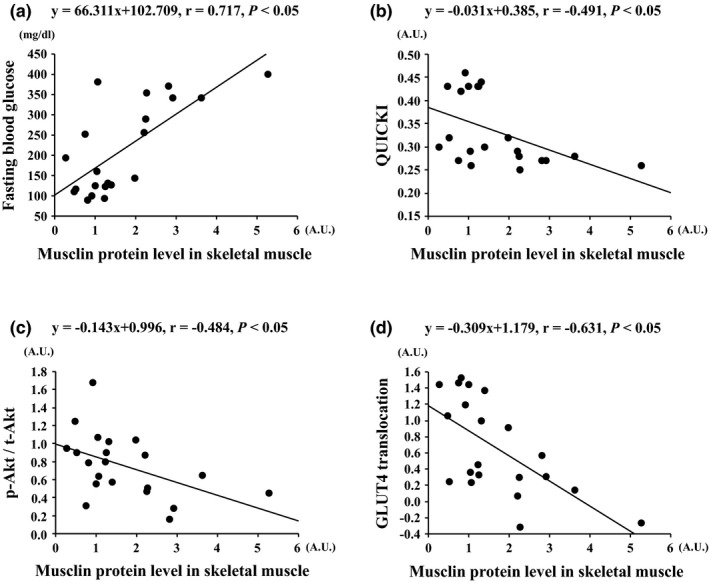
Association between the musclin protein levels in skeletal muscle and fasting blood glucose (a), QUICKI (b), Akt phosphorylation, (c) and GLUT‐4 translocation (d) levels in all groups. A.U., arbitrary units. ●, individual values

Musclin protein levels in skeletal muscle were positively correlated with fasting blood glucose (*r* = 0.717, *p* < 0.01, Figure 3a) and negatively correlated with QUICKI (*r* = −0.491, *p* < 0.05, Figure 3b), muscle Akt phosphorylation (*r* = −0.484, *p* < 0.05, Figure 3c), and GLUT‐4 translocation levels (*r* = −0.631, *p* < 0.01, Figure 3d). Additionally, musclin protein levels in skeletal muscle tended to be positively correlated with insulin levels (*r* = 0.381, *p* = 0.088).

## 4 DISCUSSION

In this study, we showed that chronic resistance exercise reduced the expression levels of musclin in the skeletal muscle of type 2 diabetes model rats with increased musclin expression and concomitantly increased muscle GLUT‐4 translocation and Akt phosphorylation. Furthermore, chronic resistance exercise decreased fasting blood glucose and reduced QUICKI, the insulin sensitivity index, in type 2 diabetes model rats. Importantly, these changes in musclin levels in the skeletal muscle were significantly associated with the fasting glucose level, the insulin sensitivity index, Akt phosphorylation, and GLUT‐4 translocation. Therefore, these results suggest that the chronic resistance exercise‐induced reduction of musclin in skeletal muscle may be important to achieve improvement of hyperglycemia and insulin resistance via the activation of the muscle Akt/GLUT‐4 signaling pathway in rats with type 2 diabetes.

Musclin inhibits muscle insulin‐stimulated Akt mRNA expression and Akt phosphorylation (Liu et al., 2008) and suppresses insulin‐stimulated glucose uptake in cultured C2C12 myocytes (Nishizawa et al., 2004). Furthermore, in obese rats fed a high‐fat diet, muscle musclin levels were positively correlated with fasting blood glucose, serum insulin levels, and the insulin resistance index (Chen, Liu, Sui, Yang, et al., 2017; Chen, Liu, Sui, Zhang, et al., 2017). In this study, a negative association between musclin expression and QUICKI, Akt phosphorylation, and GLUT‐4 translocation was observed in rats with type 2 diabetes. These results, together with the results of previous studies, suggest that chronic resistance exercise may improve the risks of type 2 diabetes mellitus with Akt activation via the reduction of musclin expression.

In this study, the basal level of the muscle‐derived secretory factor “musclin” was reduced by an 8‐week resistance exercise program in rats with type 2 diabetes, and its reduction was associated with an augmentation in GLUT‐4 translocation. Additionally, Yu et al. (2016) showed that 8 weeks of aerobic exercise reduced the basal levels of musclin expression with increased GLUT‐4 expression in the skeletal muscle of obese rats fed a high‐fat diet. Thus, musclin is reduced in both aerobic and resistance exercise modes and may be involved in the regulation of muscle GLUT‐4 via the same mechanisms. In many previous studies, not only aerobic exercise but also resistance exercise was shown to be an effective exercise mode for type 2 diabetes (Snowling & Hopkins, 2006; Umpierre et al., 2011; Yang et al., 2014). As a common mechanism of these effects of aerobic and resistance exercises, the activation of the AMP‐activated protein kinase (AMPK) and Akt signaling pathways in skeletal muscles was showed to be involved in the regulation of muscle GLUT‐4 (Kido et al., 2016). Therefore, the results of this study suggest that musclin, a muscle‐derived endocrine factor, as well as AMPK and Akt, may be common in these exercise modes involved in the regulation of muscle GLUT‐4. Thus, musclin may be a new target for the treatment of type 2 diabetes through exercise therapy.

In this study, chronic resistance exercise reduced the basal expression levels of musclin in the skeletal muscle of rats with type 2 diabetes. The mechanism by which musclin was reduced in this study is unclear. Yasui et al. (2007) showed that musclin mRNA expression in the muscle was reduced by the overexpression of forkhead box O1 (Foxo1) gene expression in the gastrocnemius muscle of skeletal muscle‐specific Foxo1 transgenic mice. Acute resistance exercise in obese patients with insulin resistance elevates Foxo1 mRNA expression levels in the skeletal muscle (Sullivan et al., 2020). In addition, acute concentric muscle contraction exercise transiently induces an increase in the mRNA expression level of Foxo1 in skeletal muscle, which then returns to basal levels (Nedergaard et al., 2007; Stefanetti et al., 2014). Since the climbing exercise used in this study requires concentric muscle contraction, the increase in Foxo1 expression by the acute climbing exercise may have downregulated the transcriptional regulation of musclin. Thus, repeating this response via acute climbing exercise may affect basal musclin expression in the skeletal muscle.

This study has some limitations. First, the correlations in this study did not clarify cause and effect. Thus, further studies are needed to examine the effect of chronic resistance exercise with exogenous administration of recombinant musclin on hyperglycemia, insulin resistance, and the muscle GLUT‐4 signaling pathway in rats with type 2 diabetes. Second, it is necessary to confirm the effect of chronic resistance exercise on muscle musclin expression in healthy rats. Third, further investigations may be required to examine the effect on musclin expression in non‐active muscles during resistance training of the other leg as a negative control by using one‐leg electrical stimulation (Ogasawara et al., 2014).

In conclusion, chronic resistance exercise in rats with type 2 diabetes reduced the expression levels of musclin in the skeletal muscle and concomitantly improved hyperglycemia and the insulin sensitivity index by activating Akt/GLUT‐4 regulated signaling in skeletal muscle. Furthermore, these changes in musclin levels in the skeletal muscle were significantly associated with fasting glucose levels, the insulin sensitivity index, Akt phosphorylation, and GLUT‐4 translocation. Therefore, these results suggest that the chronic resistance exercise‐induced reduction of musclin is involved in the improvement of hyperglycemia and insulin resistance via the activation of the muscle Akt/GLUT‐4 signaling pathway in rats with type 2 diabetes.

## CONFLICT OF INTEREST

The authors declare no conflicts of interest.

## AUTHORS’ CONTRIBUTION

Naoki H. and M.I. conceived and designed the study; M.S., Naoki H., S.F., Kenichiro I., Natsuki H., Keiko I., M.U., and M.I. performed the experiments; M.S., Naoki H., S.F., and Kenichiro I. analyzed the data; M.S., Naoki H., S.F., and M.I. interpreted the results of the experiments; M.S., Naoki H., S.F., and M.I. prepared the tables and figures; M.S., Naoki H., and M.I. drafted the manuscript; M.S., Naoki H., S.F., Kenichiro I., Natsuki H., Keiko I., M.U., and M.I. edited the manuscript; M.S., Naoki H., S.F., Kenichiro I., Natsuki H., Keiko I., M.U., and M.I. approved the final version of the manuscript.
